# Rat-atouille: A Mixed Method Study to Characterize Rodent Hunting and Consumption in the Context of Lassa Fever

**DOI:** 10.1007/s10393-016-1098-8

**Published:** 2016-02-19

**Authors:** Jesse Bonwitt, Ann H. Kelly, Rashid Ansumana, Schadrac Agbla, Foday Sahr, Almudena Mari Saez, Matthias Borchert, Richard Kock, Elisabeth Fichet-Calvet

**Affiliations:** 1 Department of Anthropology, University of Durham, Dawson Building, South Road, Durham, DH1 3LE UK; 2 Department of Sociology, Philosophy and Anthropology, University of Exeter, Exeter, UK; 3 London School of Hygiene and Tropical Medicine, London, UK; 4 Mercy Hospital Research Laboratory, Bo, Sierra Leone; 5 Medical Research Council Unit, Fajara, Gambia; 6 University of Sierra Leone, Freetown, Sierra Leone; 7 Institute for Tropical Medicine and International Health, Charité, Berlin, Germany; 8 Department of Pathology and Pathogen Biology, Royal Veterinary College, London, UK; 9 Department of Virology, Bernhard-Nocht Institute of Tropical Medicine, Hamburg, Germany

**Keywords:** Lassa fever, human–rodent interaction, *Mastomys natalensis*, hunting, consumption, mixed method

## Abstract

Lassa fever is a zoonotic hemorrhagic illness predominant in areas across Nigeria, Sierra Leone, Guinea, Liberia, and southern Mali. The reservoir of Lassa virus is the multimammate mouse (*Mastomys natalensis*), a highly commensal species in West Africa. Primary transmission to humans occurs through direct or indirect contact with rodent body fluids such as urine, feces, saliva, or blood. Our research draws together qualitative and quantitative methods to provide a fuller and more nuanced perspective on these varied points of human–animal contact. In this article, we focus on the hunting, preparation, and consumption of rodents as possible routes of exposure in Bo, Sierra Leone. We found that the consumption of rodents, including the reservoir species, is widespread and does not neatly tally against generational or gender lines. Further, we found that the reasons for rodent consumption are multifactorial, including taste preferences, food security, and opportunistic behavior. We argue that on certain topics, such as rodent consumption, establishing trust with communities, and using qualitative research methods, is key to investigate sensitive issues and situate them in their wider context. To conclude, we recommend ways to refine sensitization campaigns to account for these socio-cultural contexts.

## Introduction and Purpose

Lassa fever (LF) is a viral zoonotic hemorrhagic illness endemic in parts of West Africa with repeated outbreaks recorded in Sierra Leone, Liberia, and Nigeria (Richmond and Baglole 2003; Senior 2009; Fichet-Calvet 2014; Shaffer et al. 2014). Few cases occur regularly in Guinea, with seroprevalences up to 40% on the border with Sierra Leone (Lukashevich et al. 1993; Bausch et al. 2001). While the majority of cases are mild, presenting with non-specific signs that are difficult to distinguish from other diseases, severe cases (approximately 20% of all infections) progress to vomiting, diarrhea, pharyngitis, joint pains, and hemorrhage (Monath et al. 1974a; McCormick et al. 1987; Bausch et al. 2001; Khan et al. 2008; Asogun et al. 2012). The overall case fatality ratio is thought to be between 1 and 2%, and transient or irreversible deafness occurs in about 20% of all infections (McCormick and Fisher-Hoch 2002).

The natural reservoir of Lassa virus (LASV) is the multimammate mouse (*Mastomys natalensis*) (Monath et al. 1974b; Lecompte et al. 2006). Rodent-to-human transmission can occur indirectly, through inhaling virus-laden particles or touching food or surfaces contaminated with rodent fluids including urine, saliva, and feces or following direct contact with rodent fluids. Secondary human-to-human transmission occurs through contact with bodily fluids or contaminated objects typically in the household or in health care facilities. There is no vaccine, and prevention is recommended through hygiene improvement (food storage, rodent proofing, barrier nursing).

The social ecology of rodent-borne disease transmission has been explored with regard to housing design, agricultural practices, and consumption of meat from wild animals (Bonner et al. 2007; Taylor et al. 2008; Subramanian 2012). In Guinea, two key quantitative investigations have sought to describe the relationship between hunting and consumption of rodents and the risk of contracting LF (Ter Meulen et al. 1996; Kernéis et al. 2009). These studies found large variations in the prevalence of hunting and consumption of rodents (0–95%) and did not find an association between these activities and LF incidence in the area. However, the different time scales between the IgG serology (as a proxy of LF infection), which showed a cumulative serology over several years, and hunting activities undertaken over several weeks could be an explanation of why no association was found between the two parameters. The consumption of rodents was equally distributed across all age groups, and the majority of respondents reported only rare or occasional consumption (Kernéis et al. 2009). These findings contrast with a previous study showing that rodents are frequently captured inside houses and that all individuals irrespective of age admitted to consuming rodents (Inapogui et al. 2007). In Sierra Leone, a separate knowledge, attitude, and practice survey on LF in Kenema district (Eastern Province) revealed that 8.3% of people consumed rats after killing them, whereas 91.5% threw them away or buried them (Merlin 2002a), but further qualitative investigation from the same organization did not explore reasons behind rodent consumption (Merlin 2002b). A broader literature search on wild meat consumption indicates that large rodents are commonly hunted in western and central Africa (Fa et al. 2006; Davies et al. 2007; Subramanian 2012; Dufour 2013), but no information is available regarding small rodents species or the context within which these activities take place.

The simultaneous occurrence of various potential risk factors for LF makes hunting and consumption of rodents difficult to evaluate in terms of risk for contracting LF. Given that infected animals shed LASV in urine and blood (Monath 1975; Walker et al. 1975), it is likely that exposure to reservoir fluids, particularly during killing and butchering of infected animal serves as a pathway to infection. In this study, we used a combination of qualitative and quantitative approaches to investigate the hunting and consumption patterns of rodents and thus develop a more nuanced appreciation of this domain of human–rodent interaction. Qualitative methods, which privilege an open-ended, flexible, and iterative approach, can help shed light on practices that run counter to public health messages. Informal conversations and observations of people’s behavior can further help illuminate the discrepancies between people’s ideas and reported behavior with their actual preferences and activities and contribute to more robust prevention strategies and sensitization campaigns. Quantitative methods privilege large numbers of persons, on which trends in behavior can be supported statistically. The study was conducted in Bo district, Sierra Leone, an endemic LF area (Shaffer et al. 2014). Our work on human–rodent interactions is part of a wider eco-epidemiological study to understand the interactions between human behaviors, rodent ecology, and disease incidence in humans.

## Methods

### Study Site

The population of Sierra Leone is composed of more than 18 different ethnic groups, belonging to distinct language groups. While socio-political confederations may cut across linguistic lines with much ‘creolisation,’ the majority group is the Mende (32.2%), closely followed by the Temne (31.8%). A majority of the population (60%) is rural with a literacy rate of 43.3% (SSL 2007; CIA 2014) and just over half of the population lives below the poverty line (World Bank 2014).

The study was conducted between May and June 2014 (8 weeks) in Bo district in the Southern Province of Sierra Leone. In this district, the Mende are the dominant ethnic group (79%), followed by the Temne (7%), with Islam (72%) and Christianity (27%) the principle religions (SSL 2010). In rural areas, farming, fishing, and hunting serve as means of subsistence or to generate cash income.

### Qualitative Component

The qualitative component of this study consisted of in-depth semi-structured interviews (IDI, *n* = 21), informal discussions, focus group discussions with ad hoc recruited participants (FGD, *n* = 4), and direct observations over the entire duration of the study time. Fourteen villages were purposively selected to include those of varying size, location, and distance from main transport axes. Between 1 and 3 discussions (IDI and/or FGD) were done in each village. Selection of villages was restricted according to travel distance for the study team (max = 40 km). Individuals were chosen purposefully to achieve representation from various groups (socio-economic status, religion, ethnic group, age, sex), those knowledgeable of the community (chief, teachers), and those known to engage in the behaviors of interest. These people were identified making use of the long-standing and continuous presence of our local research team in the area since 2010. Discussions were carried out in Mende, Krio, or English and facilitated by a translator using guidelines for IDI/FGD translations (Oxfam 2012). Prompts were adapted in an iterative process to inform new data collection and were supplemented with photographs of rodent species. The prompts relevant to this report covered food security, knowledge of rodents (e.g., vernacular names, habitats, morphology, and behavior), specific interactions with rodents (e.g., avoidance, hunting, preparation, and consumption), and knowledge of LF (e.g., transmission routes, symptoms, and prevention). Prompts were refined midway during the fieldwork as new themes emerged and included new topics on LF sensitization messages and attitudes toward our research. Interviews lasted on average for 1 h and were conversational and open-ended, treated as occasions for a mutual exchange of information rather than an opportunity to extract specific data. The research team devoted as much time as possible to informal interactions with the communities to establish trust. IDIs and FGDs were recorded and transcribed, and observations were documented with field notes and photographs. For analysis, transcripts were reviewed on a daily basis using a narrative analysis, focusing on the ways in which experiences of rodent interactions were relayed, their emotional content and temporal structure and thematic analysis, drawing out repeating motifs between the responses. A priori codes corresponding to biomedical risk factors for disease transmission (e.g., procedures for handling live and dead rodents) were developed prior to fieldwork. Because of the paucity of previous research, themes related to the wider socio-economic context of human–rodent interactions were generated using emergent codes. These were further discussed with the translators to verify that interpretive categories were correct. Text segments were then color-coded according to the categories of interest.

### Quantitative Component

A cross-sectional questionnaire survey was carried out midway during the fieldwork by local staff based on findings from the qualitative component. Questions were in English with use of Mende terms and answer format was either single choice, multiple choice, or open-ended. Open-ended answers were used to determine any emerging themes that could inform the qualitative component. The quantitative survey contained 55 questions covering all forms of contact with rats (contact in homes, contact during hunting, butchering, and consumption) as well as food security and knowledge of LF. The survey was conducted with smartphones using OpenDataKit software (http://opendatakit.org), automatically collated on formhub (https://formhub.org) and exported as Microsoft Excel files.

The quantitative survey was carried out in nine villages selected by convenience as described above, with population size ranging from 500 to 1500. Because village populations varied in size, we initially intended selection with a constant sampling fraction (6%) to determine the sample size per village. However, this approach was abandoned midway through the survey when Ebola virus disease (EVD) was confirmed in the neighboring district, thus not all villages have the same proportional representation. Selection of individuals was carried out according to the WHO EPI Coverage Survey method (WHO 2008) due to the unavailability of a sampling frame at village level. A maximum of two individuals were surveyed per household, alternating between adult male, adult female (>18 years old), young male, and young female (<18 years old). Visits were done in the morning or evening as this is when most of the villagers are present and available.

In total, 524 subjects were recruited. Seven records were excluded because no village name was indicated, 57 because respondents lived in a major city (no comparative qualitative work was done in urban areas), 20 because respondents lived in other villages than the nine selected villages, and 11 because missing data on at least one outcome. The final sample size was thus 429. Sample size varies by question because skips were used to avoid asking redundant or irrelevant questions. Records with answers stating “unknown” or “don’t know” were not included in the analysis for that particular question. We provided a simple statistical description of the study participants from all nine villages. We calculated actual sampling fractions for each village, which we used as weights to account for differences in probability of selection. Accounting for the sampling design by Taylor linearization, we estimated proportions of subjects with respect to knowledge of Lassa fever, rats hunting, rats preparation, and rats consumption. We then carried out univariable and multivariable logistic regression models on each of the following outcomes: “hunted rats in the past 3 months,” “ever hunted rats,” “prepare rats at present,” “ate rats in the past 3 months,” and “ever eaten rats.” The explanatory variables used are “think that eating rats can cause disease (yes, no),” gender (female, male), age group (5–14, 15–24, 25–39, 40 years or above), educational level (none, primary, secondary or above, other), ethnicity (Mende, other), and religion (Muslim, Christian). No model selection approach was used. Adjusted Wald tests were used to assess whether there is evidence of association between explanatory variable and outcome. All analyses except the description of the study participants were performed with finite population correction using villages as strata, and were conducted with STATA 13. (StataCorp. 2013, TX: StataCorp LP).

The study was approved by the ethics committee of the Government of Sierra Leone, Charité Berlin, and the Royal Veterinary College. An informed consent form was read out in English, Mende, or Krio to each participant, and consent was obtained from all individual participants included in this study. At the end of every visit, villagers were given a specific opportunity to ask questions about LF.

## Results

The socio-demographic characteristics of the study participants in the quantitative survey are given in Table 1. None of the informants approached refused to participate in the study. In the following text, “informant” refers solely to results derived from qualitative survey during discussions and observations.

**Table 1:** Socio-demographic Characteristics of Study Participants (Quantitative Survey).

Characteristics	Number of recruited subjects, *n* (%)
Overall	429 (100)
Gender	
Female	232 (54.1)
Male	197 (45.9)
Age group (years)	
5–14	63 (14.7)
15–24	91 (21.2)
25–39	139 (32.4)
40 or above	136 (31.7)
Educational level	
None	147 (34.3)
Primary	111 (25.9)
Secondary or above	74 (17.3)
Other^a^	97 (22.6)
Ethnicity	
Mende	385 (89.7)
Other	44 (10.3)
Religion	
Muslim	334 (77.9)
Christian	95 (22.1)

^a^Usually refers to Koranic schooling.

### Terminology

During discussions, nearly all informants were able to correctly identify and name individual species from photographs. Species are distinguished, and sometimes named, according to their physical characteristics (color, markings, hairiness, size, shape, smell), their behavior (diet, nocturnal or diurnal), or the location where they are found (house, village, bush, swamp). Shrews (*Crocidura* spp.) and small- to medium-sized rodents (such as *Lemniscomysstriatus*, *Lophuromyssikapusi*, *Musmusculus*, *Nannomys* spp., *Rattus* spp., *Praomys* spp., *Mastomys* spp.) are collectively termed “rats” in English, “arata” in Krio, and “nyini” in Mende. In our study, we use the same categorization when referring to the word “rat.” Larger species of rodents, such as the cane rat (*Thryonomys swinderianus*) and the Gambian pouched rat (*Cricetomysgambianus)* do not fit into this category. Individual species are referred to using their vernacular name in Mende (Table 2). Nevertheless, *Mastomys* spp. (*M. erythroleucus* and *M. natalensis*) and *Praomys* spp. (*P. rostratus* and *P. tullbergi*) are morphologically very similar, especially when observed at dusk, the peak activity time for both species (Duplantier and Granjon 1992), hence these species are not distinguished and together are termed “vorley.” Overall, species can also be grouped into “bush rats” (e.g., *Lemniscomysstriatus*, *Lophuromyssikapusi*, *Mastomys spp*., *Praomys* spp.) or “town/village rats” (*Mastomys* spp., *Rattusrattus*, *Musmusculus*), although these are flexible categories that vary according to where the animal is mostly seen. *M. natalensis* is considered both a bush rat and town rat as it is confused with either *M. erythroleucus* (a bush rat), *Praomys* spp. (a bush rat), or *Rattusrattus* (a town rat). Our ongoing ecological studies indicate that *M. natalensis* and *R. rattus* share the commensal habitat in rural villages around Bo (mean ratio of *M. natalensis* to other commensal rodents: 60%, 222/373, range 25–84%, unpublished data).

**Table 2:** Vernacular Name of Rodent and Shrew Species in Mende.

**Foogbete** *Lemniscomys striatus* (typical striped grass mouse) named for its diurnal behavior (foi: day)
**Gboigboi** *Lophuromys sikapusi* (brush furred mouse), named after its red color
**Gowe** *Mus musculus* (domestic mouse)
**Jukui** unidentified species—large arboreal rodent
**Kiwi** *Cricetomys gambianus* (Gambian pouched rat)
**Lindie** *Nannomys* spp. (pygmy mouse)
**Nyini** general name for small- and medium-sized rats
**Seiweh** *Thryonomys* spp. (cane rat)
**Tondui** *Rattus* spp. (black or brown rat)
**Tuli** *Crocidura* spp. (musk shrew)
**Vorley** *Mastomys* spp. (multimammate mouse) and *Praomys* spp. (soft-furred mouse)

### Knowledge of LF

Most informants had previously heard of LF and considered it a serious and fatal disease, expressing familiarity with the symptoms and the special burial practices required for deceased cases (such as the use of body bags and not touching the deceased during burials). For the quantitative survey, less than half of respondents associated LF with animals (38.3%, 173/429, Table 3) and during discussions none of the informants knew the exact LF reservoir but frequently mentioned that shrews in particular could transmit LF.

**Table 3:** Results from Qualitative and Quantitative Surveys.

	Qualitative		Quantitative	
	No of recruited subjects (*n*/*N*)	Proportion (%)	No of recruited subjects (*n*/*N*)	Estimated proportion—(95% CI)^a^
A: knowledge of Lassa fever				
Rats consumption can cause disease	20/21	95	195/429	43.4 (38.1–48.8)
Have heard of Lassa fever	24/24	100	350/429	81.2 (76.2–85.4)
Know how Lassa fever is transmitted				
Contaminated food			84/429	20.3 (16.0–25.4)
Humans			40/429	9.0 (6.3–12.9)
Animals			173/429	38.3 (33.1–43.8)
B: rodent hunting				
Know anyone else who hunts rats	21/22	95	153/429	35.0 (30.3–40.0)
Hunted or caught rats in the last 3 months	8/17	47	54/429	11.4 (8.7–14.9)
Ever hunted or caught rats	18/19	95	186/429	42.4 (37.1–47.9)
Touch live rats during hunting	7/7	100	125/186	69.0 (61.2–75.9)
Ever been bitten during hunting	4/9	44	53/186	28.0 (21.5–35.6)
Ever been urinated on during hunting	2/3	66	61/186	32.2 (24.8–40.6)
C: preparation for consumption				
Prepare rats for eating at present	5/15	33	189/429	47.8 (42.3–53.4)
Come into contact with blood or guts during preparation	4/4	100	188/189	99.6 (97.2–99.9)
Wash hands after preparation	0/2	0	138/189	67.0 (59.1–74.0)
D: rodent consumption				
Know anyone who eats rats	21/23	91	97/429	20.4 (17.0–24.3)
Ate rats in the last 3 months	7/17	41	49/429	11.0 (7.9–15.2)
Ever eaten rats	19/20	95	318/429	75.5 (70.3–80.1)
Eat all types of rats	2/16	12.5	12/318	3.5 (2.0–6.2)

Note that skip logic (skipping certain questions according to previous answers) was used to avoid asking redundant or non-relevant questions; thus *n* varies with questions.

^a^These proportions are obtained after accounting for the sampling design. They estimate proportions in the total population of the 9 villages recruited into the study.

The reasons for this belief are multifactorial: shrews, whether caught in the town or in the bush, are deemed “different from other rats,” in terms of their behavior (aggressiveness), diet (carnivorous), and morphology (musk gland, elongated snout), an understanding that may have been compounded by errors in the delivery and/or comprehension of previous sensitization messages citing shrews as the reservoir for LASV. Similarly, town rats also have a “repellent” morphology (poor coat condition, maggots, on skin) and are seen to live in unhealthy places that are in close proximity to humans (such as in toilets, cemeteries, and garbage dumps), which tends to associate this category of rat with endemic diseases such as LF, malaria, yellow fever, typhoid fever, cholera, and EVD.

### Hunting

The term “hunting” is used here to describe any form of catching or trapping of rodents, from subsistence activities to child’s play, but excludes other specific rodent control methods such as rodenticides and cats.

**Figure 1:**
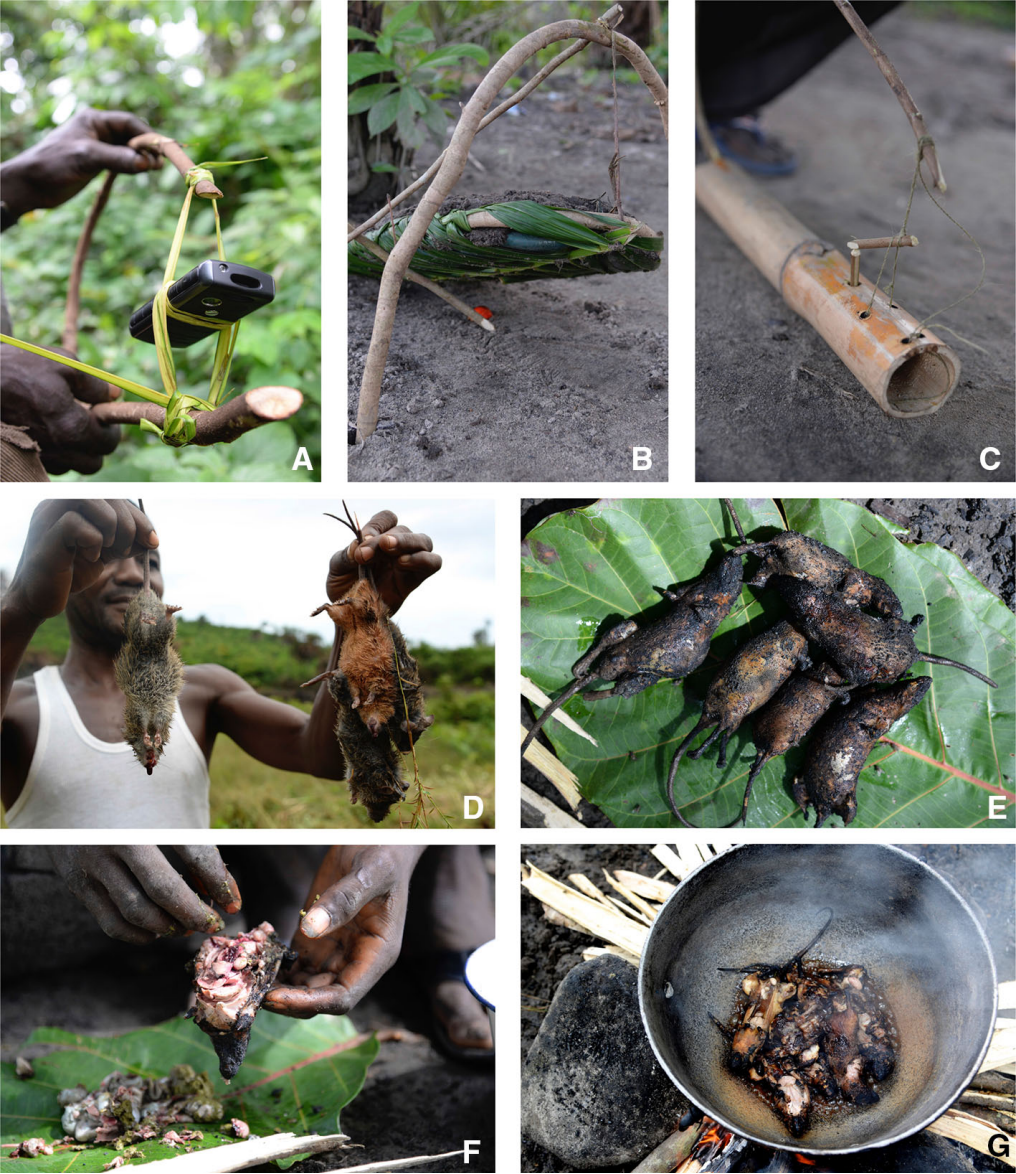
**a** Torley trap with mobile phone for scale, **b** kongoumie trap, **c** gbushie trap, **d** killed, **e** singed, **f** eviscerated, and **g** fried *L.* *sikapusi* and *Mastomys* spp.

Traps are built specifically to catch rats. “Torley” (Fig. 1a) and “kongoumie” (Fig. 1b) are trigger traps that ensnare a rat when it touches the bait. Torley is the most commonly used and easy to construct, and can be carried in bundles of up to 50 pieces. Gbushie (Fig. 1c) is a heavy clay structure that crushes the prey when it touches the bait. Traps are used mostly during the dry season because this is when rats are believed by respondents to be most active. In some villages, setting traps in the bush was described as a child’s activity, but in other villages “even the elders” use them. Rat hunting can also be done by “brushing,” an agricultural practice that refers to clearing land with machetes and that can involve multiple people (from 2 to 40 individuals) who “brush” in a circular pattern to kill rats with machetes as they are flushed out from the grass. Traps and brushing are used to kill rats for pest control or for food. A similar method for catching rats is to surround houses, kitchens, or farmhouses when they are being dismantled or repaired, which reportedly resulted in catches of 20–45 rats at a time. Brushing is a predominantly male activity, although women and children will also participate if present, or occasionally organize their own brushing. Hunting of rats by women tends to be opportunistic, for example if rats are encountered in granaries. Unlike meat from larger wildlife species, rat meat is not hunted for commercial reasons and is rarely found for sale. Direct contact with rats and their fluids can occur during any of the described forms of hunting.

**Figure 2:**
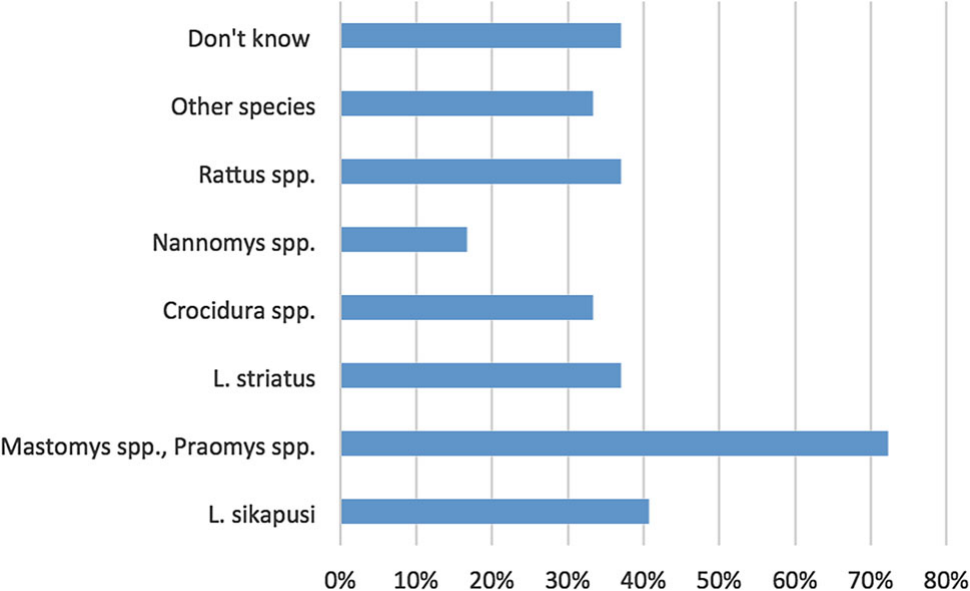
Proportion of respondents having caught various rodent species over the past 3 months (*n* = 54).

The qualitative and quantitative surveys showed that hunting activities are often practiced (Table 3). Many informants (qualitative) and respondents (quantitative) declared having hunted over their lifetime (95%, 18/19 and 42.4%, 186/429) and in the past 3 months (47%, 8/17 and 11.4%, 54/429). The quantitative survey also indicates that *Mastomys* spp. and *Praomys* spp. are the most commonly caught species (Fig. 2). Further, more than two-thirds of rat hunters handled live rats during hunting (69.0%, 125/186) and about one-third reported having been in contact with urine or having been bitten (32.2%, 61/186 and 28.0%, 53/186, respectively, Table 3).

### Consumption

Rats are singed over a fire to remove the hair, eviscerated, and sometimes butchered (Fig. 1d–g). Organs such as kidneys, liver, and heart may be kept. Rats are then smoked, grilled, and/or stewed. The meat, including bones, is eaten on its own or added to pulse dishes. No basic hygiene measures were observed during the study period despite contact with blood being reported frequently in the quantitative survey (99.6%, 188/189, Table 3). During our direct observations, nobody washed their hands, in contrast to the responses given in the quantitative survey (67%, 138/189, Table 3). Both women and men of all ages are involved in preparing rats, and these are either eaten alone or shared with friends and family depending on the size and location of the catch.

**Figure 3:**
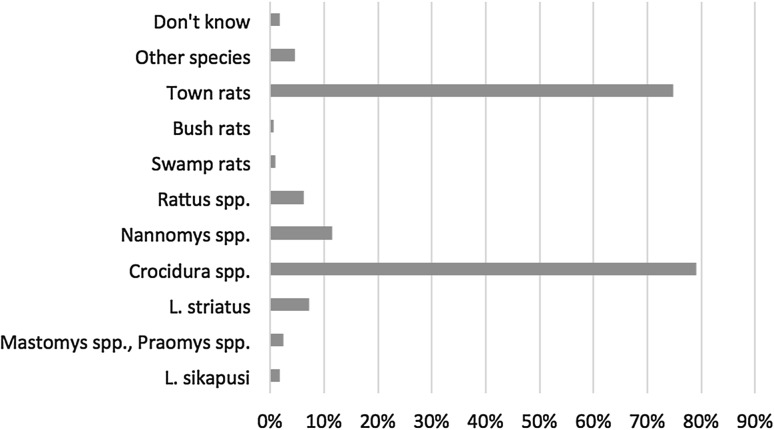
Proportion of respondents that do not eat specific rodent species (*n* = 321).

All species of rodents are eaten except for shrews (irrespective of where they are caught) and town rats (Fig. 3). Some Muslims reported not eating *L. striatus* because they interpreted the striped coat as divine writing. With very rare exceptions, adults reported never eating town rats or shrews. Those that did justified it because of hunger or as an act of defiance toward (public health) authorities. When interviewed without the presence of an adult relative, children were more readily to admit eating both bush and town rats. Parents acknowledged that their children probably hunted and ate rats in hiding, and that there was little that they could do to stop them.

Preparing and consuming rats was carried out without ceremony or occasion. A commonly cited reason for consumption was its supplementary value: rat meat provides a “very important source of protein” and is necessary for maintaining a “balanced diet”, satisfying a “want of meat”, born “out of poverty.” It is important to note that this desire for nutritional security was expressed not in terms of quantity but for a diverse quality of food sources. All informants bar two (one adult, one child) stressed that they would not go hungry if rat was no longer available. In contrast to bland foods such as rice or beans, rat meat was overwhelmingly described as ”a sweet (tasty) meat” that makes a “very good dish,” so much so that it was popularized in a song by singer Amie Kallon in the 1970s describing rat meat as “sweeter” than cow meat. About half of the informants stressed that they would continue eating rat meat even if cow meat was available and affordable. Those that expressed a preference for beef emphasized the comparatively fewer bones and more meat in cows, though some added that large rodents, which combine the benefits of both rat and cow meat, are preferable to both.

However, questions of consumption preference were ultimately trumped by pragmatism: it was considered wasteful to throw away bush rats killed for pest control. Many informants explained that while other sources of protein are available (larger wildlife species, fish, chicken, frozen fish) rat meat is free, easy, and fast to catch. Informants no longer consuming rats reported eating less meat or spending more money to buy alternatives. Among those who no longer ate rats (or said they didn’t), fear from disease, in particular LF, was by far the single most important reason. Linguistic responses by those who confirmed rat consumption included words or expressions such as “tempted,” “trying not to,” and “nearly stopped,” suggesting that whatever the reasons for eating rats, they outweighed the fear generated by LF.

Many informants talked freely and casually about rat consumption, but some would contradict themselves, modify their statements, change tense, or cut the conversation short. Other informants opened up as trust was established (“I don’t want to lie,” “I will be honest”). In addition, the proportion of behaviors reported during IDIs and FGDs contrasted with the proportion reported during the quantitative survey (Tables 3 and 4). Rat consumption was more often reported during the IDIs and FDGs.

**Table 4:** Excerpts from Discussions on Hunting and Consumption of Rodents (IDI: In-depth Interview, FGD: Focus Group Discussion).

“No, we never hunt them.” (chief, village 12, contradicted by IDI in same village)
“Everyone eats them.” (unemployed, subsistence farmer, village 14)
“I can say that in the village, almost all of us (eat rats).” (teacher, village 15)
“Everybody, even like this boy [pointing]. The kids are fond of it but everybody from childhood to adulthood, everybody eats it. Men, women children.” (chief, village 18)
“Except very few, minus the town rats, they can eat almost all the rats in the bush. If some people deny that they don’t eat it, it is something sceptical because most people who deny that they don’t eat rat if they are still eating it.” (chief, village 13)
“In this village many people eat rat but they never announced this disease in town so people do not believe it. People here eat rat every day.” (chief, village 19)
“Here if they [you] say 100, 90 per cent [of them] eat rat.” (subsistence farmer, village 26)
“Before now we are eating them. But the eating ways, or the eating habits, has been minimized. We do not rule out completely that people are not still eating it; they are eating it, but that has been minimized.” (FGD, village 27)
“We have almost stopped eating them…[it is] not completely over.” (FGD, village 27)
“Most of them [people] [eat rats].” (FGD, village 28)
“It is minimized, but if they find [rodents] in the bush they will eat” (FGD, village 28)

In the multivariate analysis, gender was the only variable that was consistently and significantly associated with a history of hunting rats in the past 3 months and over lifetime, preparation of rats, and consumption of rats in the past 3 months and over lifetime (Table 5). All other variables (age, education, religion, and ethnic group) were not significantly or consistently associated with these behaviors.

**Table 5:** History of Hunting Rodents in the Past 3 Months and over Lifetime, Preparation of Rodents, and Consumption of Rodents in the Past 3 Months and over Lifetime was Analyzed According to Sex, Age, Religion, and Ethnic Group.

Variables	Unadjusted OR—(95% CI)	*P* ^a^	Adjusted OR—(95% CI)	*P* ^a^
Hunted rats in the past 3 months				
Think that eating rats can cause disease (ref.: no)				
Yes	1.31 (0.68–2.52)	0.41	1.33 (0.64–2.76)	0.45
Gender (ref.: female)				
Male	4.46 (1.93–10.3)	0.001	5.01 (2.20–11.4)	<0.0001
Age group, years (ref.: 40 or above)		0.13^b^		0.02^b^
5–14	1.90 (0.73–4.97)	0.19	2.13 (0.63–7.26)	0.22
15–24	0.94 (0.35–2.55)	0.91	1.48 (0.47–4.74)	0.50
25–39	2.24 (0.96–5.23)	0.06	3.36 (1.49–7.58)	0.004
Educational level (ref.: none)		0.30^b^		0.27^b^
Primary	2.15 (0.83–5.55)	0.12	1.86 (0.65–5.30)	0.25
Secondary or above	1.21 (0.41–3.60)	0.73	0.91 (0.27–3.08)	0.88
Other	2.11 (0.79–5.59)	0.13	2.03 (0.77–5.32)	0.15
Ethnicity (ref.: Mende)				
Other	0.83 (0.25–2.75)	0.76	0.53 (0.16–1.87)	0.30
Religion (ref.: Muslim)				
Christian	0.68 (0.27–1.72)	0.41	0.66 (0.16–1.74)	0.43
Ever hunted rats				
Think that eating rats can cause disease (ref.: no)				
Yes	1.35 (0.85–2.14)	0.20	1.17 (0.63–2.17)	0.63
Gender (ref.: female)				
Male	9.31 (5.37–16.2)	<0.0001	9.95 (5.28–18.8)	<0.0001
Age group, years (ref.: 40 or above)		0.47^b^		0.35^b^
5–14	0.70 (0.32–1.52)	0.36	0.58 (0.20–1.69)	0.32
15–24	0.61 (0.32–1.15)	0.13	0.69 (0.30–1.59)	0.38
25–39	0.84 (0.48–1.47)	0.54	1.18 (0.56–2.47)	0.67
Educational level (ref.: none)		0.14^b^		0.37^b^
Primary	0.92 (0.50–1.70)	0.79	0.78 (0.36–1.68)	0.52
Secondary or above	2.10 (1.03–4.27)	0.04	1.87 (0.72–4.87)	0.20
Other	1.28 (0.71–2.31)	0.41	1.01 (0.50–2.03)	0.97
Ethnicity (ref.: Mende)				
Other	1.08 (0.51–2.26)	0.84	0.62 (0.28–1.39)	0.24
Religion (ref.: Muslim)				
Christian	0.86 (0.47–1.57)	0.62	0.84 (0.38–1.88)	0.67
Prepare rats at present				
Think that eating rats can cause disease (ref.: No)				
Yes	0.59 (0.37–0.92)	0.02	0.49 (0.30–0.80)	0.005
Gender (ref.: female)				
Male	1.81 (1.14–2.87)	0.01	2.19 (1.34–3.57)	0.002
Age group, years (ref.: 40 or above)		0.31^b^		0.21^b^
5–14	1.63 (0.78–3.42)	0.20	1.45 (0.63–3.37)	0.39
15–24	1.57 (0.84–2.92)	0.16	1.89 (0.95–3.75)	0.07
25–39	1.59 (0.90–2.79)	0.11	1.77 (0.97–3.25)	0.06
Educational level (ref.: none)		0.19^b^		0.23^b^
Primary	0.76 (0.42–1.39)	0.38	0.68 (0.34–1.35)	0.27
Secondary or above	1.58 (0.88–2.86)	0.13	1.39 (0.73–2.65)	0.32
	0.94 (0.52–1.68)	0.83	0.90 (0.48–1.68)	0.75
Ethnicity (ref.: Mende)				
Other	0.49 (0.21–1.18)	0.11	0.51 (0.20–1.27)	0.15
Religion (ref.: Muslim)				
Christian	1.57 (0.87–2.86)	0.14	1.33 (0.74–2.38)	0.34
Consumed rats in the past 3 months				
Think that eating rats can cause disease (ref.: no)				
Yes	0.77 (0.38–1.58)	0.48	0.71 (0.27–1.86)	0.48
Gender (ref.: female)				
Male	2.70 (1.19–6.14)	0.02	2.68 (1.12–6.42)	0.03
Age group, years (ref.: 40 or above)		0.15^b^		0.22^b^
5–14	5.17 (1.11–24.1)	0.04	4.34 (0.71–26.6)	0.11
15–24	2.16 (0.49–9.49)	0.31	2.41 (0.47–12.3)	0.29
25–39	3.00 (0.73–12.3)	0.13	3.77 (0.99–14.3)	0.05
Educational level (ref.: none)		0.02^b^		0.21^b^
Primary	3.22 (1.31–7.89)	0.01	2.16 (0.67–6.99)	0.20
Secondary or above	3.30 (1.22–8.92)	0.02	2.36 (0.62–8.98)	0.21
Other	2.73 (1.08–6.93)	0.03	2.71 (0.93–7.87)	0.07
Ethnicity (ref.: Mende)				
Other	1.70 (0.43–6.74)	0.45	1.58 (0.41–6.16)	0.51
Religion (ref.: Muslim)				
Christian	1.37 (0.62–3.03)	0.44	1.18 (0.55–2.55)	0.67
Ever consumed rats				
Think that eating rats can cause disease (ref.: No)				
Yes	1.66 (0.98–2.82)	0.06	1.36 (0.75–2.47)	0.30
Gender (ref.: female)				
Male	2.67 (1.51–4.70)	0.001	3.39 (1.87–6.17)	0.0001
Age group, years (ref.: 40 or above)		0.24^b^		0.09^b^
5–14	0.77 (0.34–1.74)	0.54	0.47 (0.19–1.19)	0.11
15–24	1.74 (0.88–3.46)	0.11	1.38 (0.61–3.16)	0.44
25–39	1.20 (0.60–2.38)	0.61	1.28 (0.60–2.72)	0.52
Educational level (ref.: none)		0.06^b^		0.36^b^
Primary	1.01 (0.50–2.05)	0.97	1.26 (0.60–2.66)	0.55
Secondary or above	2.36 (1.11–5.03)	0.03	1.94 (0.81–4.67)	0.14
Other	0.82 (0.42–1.62)	0.57	0.86 (0.43–1.70)	0.67
Ethnicity (ref.: Mende)				
Other	0.30 (0.15–0.62)	0.001	0.21 (0.09–0.46)	0.0001
Religion (ref.: Muslim)				
Christian	1.63 (0.73–3.63)	0.23	1.44 (0.62–3.37)	0.40

^a^Adjusted Wald test assessing the significance of estimated odds ratio.

^b^Adjusted joint Wald test assessing the association between the related explanatory variable and outcome, which is needed for categorical explanatory variables.

### Reluctance to Talk About Rat Consumption

In response to the clear discomfort that the topic of rat consumption generated for some of our informants, interview prompts were refined to explore the reasons for that reticence. A recurrent theme was the fear of talking about rat consumption to strangers. Informants explained that they were afraid to acknowledge this practice because they had been advised against eating rats through sensitization messages from health care workers and through the radio. The survey team aroused suspicion, as they were identified as government workers, taking notes on electronic devices to send to “higher authorities.” It was thought that if authorities knew about who ate rats, they might prevent those persons from accessing health care services, take them away for testing, or even inject them with LASV. Other reasons were also mentioned, such as fear of blackmail (from the research team), and being excluded from potential benefits that the study might bring. However, there was no sense of shame in admitting to eat bush rats, in contrast to town rats and shrews.

## Discussion

The study was conducted in one rural district of Sierra Leone with a predominantly Mende population, thus the findings cannot be considered representative of the whole country. However, we believe that some of its findings are likely to have broader regional relevance across the Mano River basin, as Mende, Kissi, Kono, Toma/Loma frequently mix, migrate, and thus share a history and culture (Fairhead and Leach 1996).

There was a strong consensus regarding the methods employed for hunting, preparing, and cooking rats. Contact with rodent fluids (blood, urine, saliva via biting) was commonly reported and is likely to pose a risk for zoonotic transmission of LF given the presence of LASV in various bodily fluids and organs of *M. natalensis* (Monath et al. 1974b; Walker et al. 1975), and the likely high prevalence of LASV in *M. natalensis* in the region (Fichet-Calvet and Rogers 2009). Although nearly three quarters of people reported washing their hands after manipulating rats, it is unlikely that disinfection occurs given the lack of sanitary options on the farms. Therefore, this result should be considered critically, especially given the contradictory results provided by the direct observations and IDIs. Further, rodents are one of the taxonomic groups most associated with emerging infectious diseases (EID) in humans (Wolfe et al. 2007; Han et al. 2015). The observed high frequency of contact with rodent fluids (especially during butchering) in our study area argues for the need for risk-based surveillance systems for EIDs that can also be informed by socio-anthropological studies.

Consumption of bush rat was much more frequent than town rats and shrews, a choice overwhelmingly explained by the respective habitats and behaviors of these two categories. The significance of these contacts in terms of LASV transmission depends on their frequency and on the proportion of *M. natalensis* in the catch, i.e., on the number of *M. natalensis* caught and eaten. Ecological studies in Guinea suggest that *M.natalensis* is predominantly found in houses and proximal cultivations, but infrequently in distal cultivations (Fichet-Calvet et al. 2007), thus consumption of *M. natalensis* might be less important relative to other rodents. The potential in variation to LASV exposure along a geographically induced behavioral gradient (consumption of bush rats vs. non-consumption of town rats), as well as preference and avoidance of certain species, provides a strong argument to investigate human behaviors as drivers for disease emergence (Kock 2014) and their overlay with the eco-epidemiology of zoonotic diseases and reservoir species.

Hunting rats does not tally against specific generational, ethnic, or religious attributes; rather it is a highly opportunistic and domestic practice in which the vast majority of people engage. The quantitative survey showed that males were consistently more likely to hunt, prepare, and consume rats than females, although the latter also engage in these activities. Interviews and observations support this result and provide a possible explanation: men spend more time on the farms and thus have more opportunities and motivations (pest control) to hunt (bush) rodents. Moreover, preliminary information indicates that hunting of rats by children is considered a “boy” activity, emulating large game hunters, who are customarily men. Child hunting can operate in a highly autonomous fashion outside of parental control, an activity that is thought to be an important part of child socialization, bridging the social and ecological environments (Gavelle J., pers. com.). This form of interaction is an important factor to consider when investigating and preventing zoonotic spillover events, as for example with the West African EVD outbreak which is believed to have started during hunting by children (Mari-Sáez et al. 2014). Yet, the fact that the majority of individuals in rural communities engage in rats hunting and/or consumption suggests that sensitization messages need to consequently target a wide audience while at the same time understanding the different motivations of community members to engage in these behaviors. Hunting and consumption of rodents is motivated by a variety of factors ranging from taste to unwillingness to waste meat when it is caught as part of pest control activities. Prevention strategies for primary transmission from animal sources therefore need to take these drivers into account and provide strong arguments or incentives to overcome the advantages associated with the consumption of meat from wild animals.

Sensitization about the dangers of *Mastomys* spp. and morphologically similar species (*Rattus* spp. and *Praomys* spp.) should provide the flexibility to mitigate contact with these species only. Given the success of using photographs of rodent species during this study, we suggest that outreach teams make more use of visual materials. There is also a need to address the misconception about the reservoirs of LF; despite long-standing sensitization campaigns in the area, many incorrectly identified shrews and town rats as the reservoir. It was difficult to ascertain whether this misconception arose from errors and inconsistencies in sensitization messages, or because of unintentional or intentional misunderstanding, for example by constructing a narrative that suits people’s practices (i.e., associating distasteful shrews with LF). Skepticism and rumors about LF (possibly heightened by the EVD epidemic) was prevalent, a consequence of deep-rooted mistrust in government and healthcare services, underlining the importance of devising clear, concise, consistent, and accurate messages (Hewlett and Hewlett 2008) and the possibility of unintended consequences, for example, on food security.

Most of the results from the qualitative and quantitative components were consistent; however, the reported prevalence of hunting and consumption of rats differed between the two. The quantitative survey indicated that 11% of respondents admitted hunting or eating rats in the past 3 months, in contrast to the qualitative component suggesting that hunting and consumption is more frequent and widespread at present (41–47%), although it is problematic to make such generalizations based on a small (*n* = 25) set of semi-standardized interviews. However, it is unlikely that informants had any motivations for overestimating the prevalence of rat consumption, whereas we identified many reasons to underreport this behavior during the quantitative survey, such as distrust toward healthcare staff already described in Sierra Leone (Merlin 2002b). Furthermore, 47.8% of respondents reported presently preparing rats for consumption, but only 11.0% reported having eaten one during the same period. This idiosyncrasy tentatively suggests that consumption is underreported, especially considering that we did not find any evidence of people preparing rats but not eating them (as might be expected with the sale of rat meat).

Previous quantitative surveys in Guinea, Côte d’Ivoire, and Sierra Leone report the prevalence of rodent hunting and consumption between 0 and 98% (Ter Meulen et al. 1996; Merlin 2002b; Akoua-Koffi et al. 2006; Inapogui et al. 2007; Kernéis et al. 2009) and more specifically at 8.3% in the Eastern Province of Sierra Leone (Merlin 2002a). However, a preliminary study conducted in coastal Guinea in 2004 revealed a 56% (79/142) prevalence, specifying the time frame “at present” when asking questions (pers. obs.), whereas the other studies might reflect cumulative data since childhood, which in this survey was 75.5%. The wide variation between studies could be due to differences or bias in the study design, or alternatively could be indicative of wide-ranging practices across western Africa. In any case, these need to be interpreted critically in light of our findings on the reluctance to talk about rat consumption in LF-endemic areas.

The implementation and analysis of our survey may have introduced another source of bias: whereas independence of the research team from official initiatives was emphasized before engaging in discussions, the public health focus of the study may have generated suspicion. Not all villages were proportionately represented and convenience sampling was sometimes used, thus the results might not be representative of villages or the population. In hindsight, it would have been useful to enforce consistency between the survey and discussions to exclude any potential bias originating from the phrasing of questions. Finally, the survey team contained only one female (out of a total of 5 surveyors), which may have biased answers from female participants. With regard to the qualitative data collection, we could have concentrated on a smaller number of villages in order to gain a more finely grained description of rodent hunting and consumption. Further investigations in this area and along these lines are currently underway.

## Conclusion

In this study, we have sought to provide a description of the socio-cultural and environmental contexts within which people and rodents interact in an LF-endemic area. Our findings point to some salient features of human–rodent interactions—the roles of taste and children’s play—and other forms of rodent interactions occurring in domestic and peri-domestic spaces that demand further anthropological and epidemiological research to characterize human–rodent interactions. The discrepancies between the survey, impromptu discussions, in-depth interviews, and observational work point to the limitations of using quantitative methods to investigate sensitive topics. As such, this study underlines the importance of situating disease within the wider socio-cultural contexts in which it occurs and illustrates the value of multidisciplinary collaboration in health research and policy.
